# Genomic Insights into the Fungal Lignocellulolytic Machinery of *Flammulina rossica*

**DOI:** 10.3390/microorganisms7100421

**Published:** 2019-10-08

**Authors:** Young-Jin Park, Chang-Soo Lee, Won-Sik Kong

**Affiliations:** 1 Department of Biomedical Chemistry, Research Institute for Biomedical & Health Science, College of Biomedical and Health Science, Konkuk University, 268 Chungwon-daero, Chungju-si 27478, Korea; cslee@kku.ac.kr; 2 Mushroom Research Division, National Institute of Horticultural and Herbal Science, Rural Development Administration, 92, Bisan-ro, Eumseong-gun 27709, Korea; wskong@korea.kr

**Keywords:** basidiomycete, carbohydrate active enzyme, *Flammulina rossica*, fungi, genome

## Abstract

Next-generation sequencing (NGS) of the *Flammulina rossica* (wood-rotting basidiomycete) genome was performed to identify its carbohydrate-active enzymes (CAZymes). De novo genome assembly (31 kmer) revealed a total length of 35,646,506 bp (49.79% GC content). In total, 12,588 gene models of *F. rossica* were predicted using an ab initio gene prediction tool (AUGUSTUS). Orthologous analysis with other fungal species revealed that 7433 groups contained at least one *F. rossica* gene. Additionally, 12,033 (95.6%) of 12,588 genes for *F. rossica* proteins had orthologs among the Dikarya, and *F. rossica* contained 12 species-specific genes. CAZyme annotation in the *F. rossica* genome revealed 511 genes predicted to encode CAZymes including 102 auxiliary activities, 236 glycoside hydrolases, 94 glycosyltransferases, 19 polysaccharide lyases, 56 carbohydrate esterases, and 21 carbohydrate binding-modules. Among the 511 genes, several genes were predicted to simultaneously encode two different CAZymes such as glycoside hydrolases (GH) as well as carbohydrate-binding module (CBM). The genome information of *F. rossica* offers opportunities to understand the wood-degrading machinery of this fungus and will be useful for biotechnological and industrial applications.

## 1. Introduction

*Flammulina rossica* (edible mushroom, *Physalacriaceae*) was first identified in 1999 by Redhead and Petersen [1]. According to a previous report, *F. rossica* is one of the recently described *Flammulina* species of the Northern Hemisphere, which include *Flammulina elastica*, *Flammulina fennae*, *Flammulina ononidis*, and *Flammulina velutipes* [2]. Redhead and Petersen [1] reported that the basidiocarps of *F. rossica* are similar to those of *F. velutipes*, although they possess a very pale pileus—whitish to yellowish ochraceous. Based on their ribosomal ITS sequences, *F. rossica* was found in a large clade with *Flammulina mexicana*, *Flammulina populicola*, and *F. fennae* but not with *F. elastica* [2]. *F. rossica* is found on the trunks of *Alnus* sp., *Populus* sp., *Salix amygdaloides*, *Salix caprea*, and *Salix* sp., from July to January [3].

Enzymes involved in the synthesis and breakdown of glycoconjugates, oligosaccharides, and polysaccharides are known as carbohydrate-active enzymes (CAZymes). CAZymes are divided into 6 classes including glycosyltransferases (GTs), carbohydrate esterases (CEs), glycoside hydrolases (GHs), polysaccharide lyases (PLs), auxiliary activities (AA), and carbohydrate-binding modules (CBMs). These CAZymes are further classified into families, based on their structural and amino acid sequence similarities [4]. CAZymes are widely recognized as one of the keys to biofuel production because they play a significant role in plant cell wall degradation. Thus, CAZymes are attracting attention as an area of biotechnological and industrial application [5,6]. Basidiomycetes are capable of efficiently degrading lignocellulosic biomass, especially plant-derived lignocellulosic biomass, due to various CAZymes [5,7]. This ability allows the fungus to live in a variety of natural conditions such as wood wastes and forest residues. Wood rot fungi are usually divided into brown rot fungus and white rot fungus. In particular, white rot fungi, which account for more than 90% of wood-rotting basidiomycetes, decompose both polysaccharides and lignin, leaving the residue white or yellowish. [5,6].

We previously performed genome-sequencing of *F. velutipes*, *F. elastica*, *F. fennae*, and *F. ononidis* and reported well-developed wood-degrading machineries in their genomes based on CAZyme identification [8,9,10,11]. Similarly, genome-sequencing studies of various organisms have been performed in order to massively excavate genes that encode biomass-degrading enzymes [5,12,13]. Moreover, research on biomass-degrading enzymes in the post-genome era is a major research field for understanding wood-degrading machinery and to describe the CAZyme repertoire of fungal species.

This study aimed to determine the genome sequence of *F. rossica* and to identify biotechnologically and industrially useful CAZyme genes. The genomic information of *F. rossica*, including the genes encoding CAZymes, will help understand this fungus and will be useful for future biotechnological and industrial applications.

## 2. Materials and Methods 

### 2.1. Fungal Strain, Culture, and Genomic DNA Isolation

*F. rossica* ASI4194 was obtained from the Mushroom Research Division, National Institute of Horticultural and Herbal Science (Rural Development Administration, Eumseong-gun, Korea) and was grown at 26 °C on potato dextrose agar (PDA) for 15 days. For genomic DNA isolation, extraction buffer (100 mM NaCl, 50 mM EDTA, 0.25 M Tris-HCl, 5% SDS), 2 × CTAB buffer (2% Cetyltrimethylammonium bromide, 1.4 M NaCl, 20 mM EDTA pH 8.0, 100 mM Tris-HCl pH 8.0, 1% polyvinyl pyrrolidone), and phenol:chloroform:isoamyl alcohol (25:24:1) were added to the mycelia and mixed vigorously. Sample was centrifuged at 12,000 rpm at 4 °C for 5 min. Supernatant was mixed with 0.7 volume of isopropanol and then centrifuged for 15 min. The pellet was washed in cold 70% then dried pellet was dissolved in TE buffer containing RNase A (Qiagen, Seoul, Korea).

### 2.2. Genome Sequencing, De Novo Assembly, Gene Prediction, and Annotation

Next-generation sequencing (NGS) of the *F. rossica* genome was performed using the HiSeq 2000 platform (Illumina, Inc., San Diego, CA, USA) according to the manufacturer’s protocol. The quality of sequencing data was evaluated using FastQC [14] and was processed using Trimmomatic (version 0.32) [15] to detect sequencing adapters and bad quality reads. Quality checked reads were used for assembly using Velvet software [16]. Gene structure modeling was processed using the AUGUSTUS software [17], trained with *Laccaria bicolor*. The predicted genes of *F. rossica* were then compared with a non-redundant database (National Center for Biotechnology Information, NCBI, Bethesda, MD, USA) using DIAMOND software [18] for functional annotation.

### 2.3. Ortholog Analysis

*F. rossica* genes were analyzed and clustered into orthologous groups using OrthoFinder (version 2.3.3) software [19] with other fungal species. *A. nidulans* FGSC-A4 [20], *Botrytis cinerea* B05.10 [21], *Agaricus bisporus* var. *bisporus* H97 [22], *C. cinerea* okayama7#130 [23], *L. bicolor* S238N-H82 [24], *Lentinula edodes* [25], *C. militaris* CM01 [26], *C. neoformans* var. *grubii* H99 [27], *F. elastica* KACC46182 [9], *F. fennae* KACC46185 [10], *F. ononidis* KACC46186 [11], *F. velutipes* KACC42780 [8], *Phanerochaete chrysosporium* RP78 [12], *S. cerevisiae* S288C [28], *Neurospora crassa* OR74A [29], *S. commune* H4-8 [30], and *T. reesei* QM6a [31].

### 2.4. CAZyme and Signal Peptide Identification 

CAZymes, including those encoded by *GH*, *GT*, *PL*, *CE*, *AA*, and *CBM* genes in *F. rossica* were identified and annotated using the dbCAN meta server including the HMMER (dbCAN CAZyme domain HMM database), DIAMOND (CAZy database), and Hotpep (short conserved motifs in the PRR library database) [32]. Signal peptide prediction in CAZyme genes was carried out using the SignalP 5.0 software [33].

### 2.5. Data Access

The raw reads were deposited in the Sequence Read Archive (SRA) database at NCBI (SRR9964086).

## 3. Results and Discussion

### 3.1. De Novo Genome Assembly, Gene Prediction, and Genome Comparisons

The short reads (total of 38,390,380; 100 bp paired-end reads) were analyzed using the Trimmomatic tool [14] for quality control including adapter trimming. The resulting short reads (35,908,618 reads, >Q30) were processed for genome assembly using the Velvet assembly software (kmer-size of 17–31) [16]. The optimized assembly (31 kmer) comprised 15,546 sequence contigs with a total length of 35,645,506 bp (49.77% GC contents) and an N50 length of 48,718 bp. In ab initio gene prediction, 12,588 genes were predicted. The average gene, exon, and intron lengths were 1911, 234.67, and 68.03 bp, respectively. The optimized assembly and gene prediction of the *F. rossica* ASI4194 genome are presented in Table 1. 

Of the 12,588 predicted genes, 83.3% (10,490) had sequence similarity (0.001 > e-value) with the proteins in NCBI-NR (Table S1). The total number of genes in *F. rossica* was comparable to that of its nearest sequenced species, *F. elastica* [9], as well as to that of other basidiomycetes with a similar genome size (Table 2).

**Figure 1:**
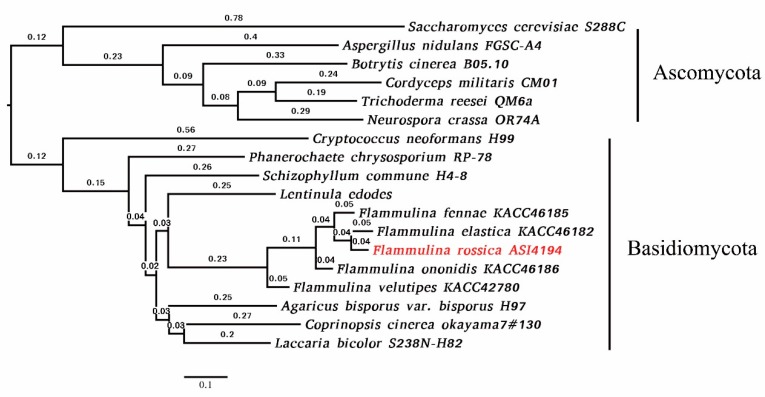
Phylogenetic tree of fungal species based on ortholog clustering.

Through cluster analysis with other fungal species, 7485 (58.7%) out of 12, 756 groups containing at least one *F. rossica* gene were identified (Table 3). In addition, 95.5% of *F. rossica* genes were conserved in the Dikarya including ascomycetes and basidiomycetes (Table 3). Among the set of homologous genes, there were 5 species-specific orthogroups containing 12 species-specific genes in *F. rossica* (Table 3). As shown in Figure 1, *F. rossica* was classified into one group with *F. elastica* and was clustered into one group together with *F. fennae*, *F. onnidis*, and *F. velutipes* by an ortholog-based clustering analysis.

### 3.2. F. rossica CAZymes and Genome-Wide Comparisons with Other Fungal Species

**Figure 2:**
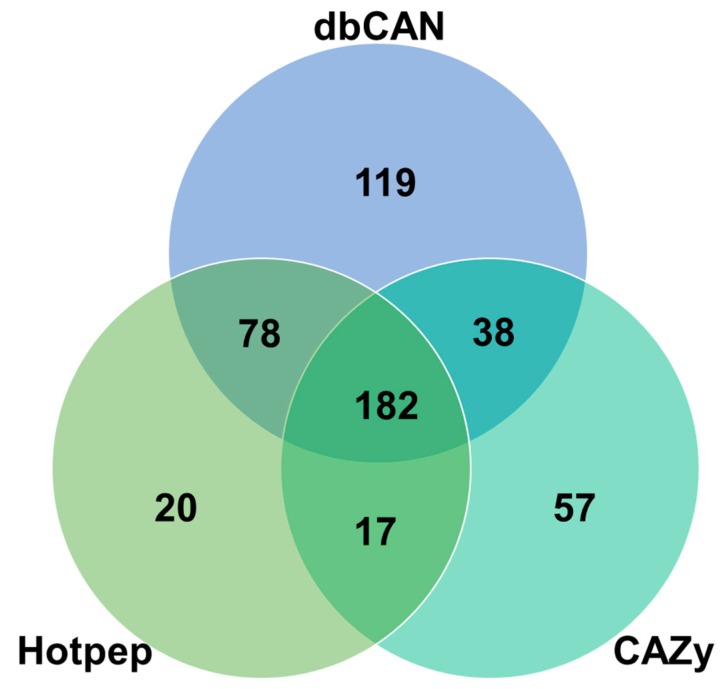
Identification and annotation of carbohydrate-active enzyme genes in the *F. rossica* genome based on three different databases including the HMMER (dbCAN carbohydrate-active enzymes domain HMM database), DIAMOND (CAZy database), and Hotpep (short conserved motifs in the (PRR library database) [32].

The genome sequence of *F. rossica* revealed a series of genes associated with the breakdown (GHs, PLs, CEs) and assembly (GT) of carbohydrate complexes. The *F. rossica* genome was also found to contain several genes encoding lignin degradation enzymes (auxiliary activities; AA) as well as a carbohydrate-binding modules (CBM). CAZyme prediction of *F. rossica* genes through the dbCAN meta server [32] including the HMMER (dbCAN CAZyme domain HMM database), Hotpep (short conserved motifs in the PRR library database), and DIAMOND (CAZy database) revealed 419, 300, and 294 CAZymes, respectively (Figure 2 and Table 4).

Among the 511 genes associated with CAZymes, several genes were predicted to simultaneously encode two different CAZymes, such as GH as well as CBM. Therefore, in total, 528 CAZymes including 102 AAs, 236 GHs, 94 GTs, 19 PLs, 56 CEs, and 21 CBMs were identified in the *F. rossica* genome (Table 4 and Table S2). For genome-wide comparison, the annotated CAZymes of eight other fungal species were obtained from the CAZy database [4] and JGI database (https://genome.jgi.doe.gov/programs/fungi/index.jsf).

#### 3.2.1. Glycosyltransferases (GTs) of *F. rossica* Genome

**Figure 3:**
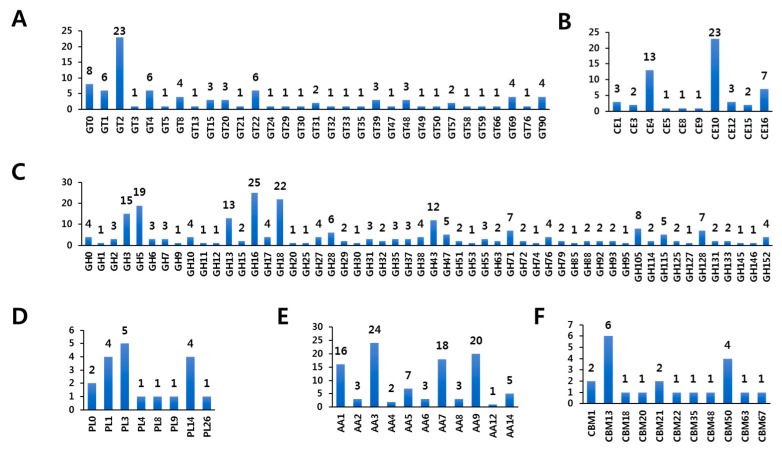
Number of CAZymes in *F. rossica*. Number of (**A**) GT families; (**B**) CE families; (**C**) GH families; (**D**) PL families; (**E**) AA families; (**F**) CBM families. AA, Auxiliary Activities; GH, glycoside hydrolase; GT, glycosyltransferase; CBM, carbohydrates-binding module; PL, polysaccharide lyase.

**Figure 4:**
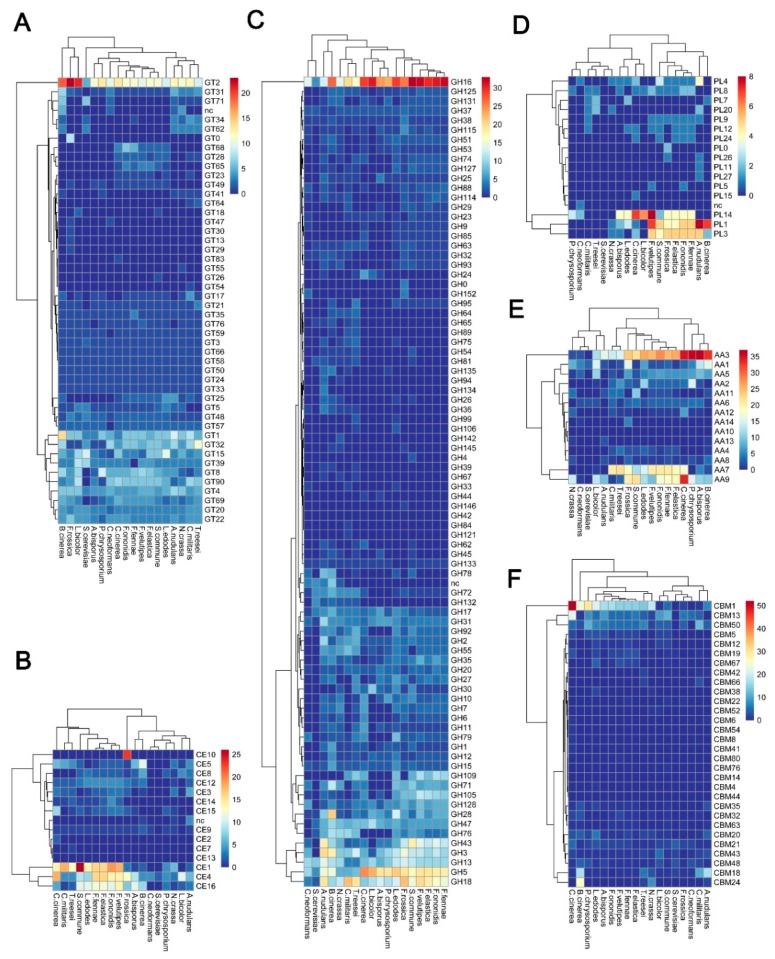
Distribution of CAZymes in *F. rossica* and other fungal species. (**A**) GT families; (**B**) CE families; (**C**) GH families; (**D**) PL families; (**E**) AA families; (**F**) CBM families.

GTs (EC 2.4.x.y) catalyze glycosyl group transfer and glycosidic linkage formation using activated donor sugar phosphates [34,35,36,37], which are involved in the biosynthesis of glycoconjugates, oligosaccharides, and polysaccharides [34,35,36,37]. CAZyme prediction revealed that *F. rossica* contains a total of 23 GT families in its genome sequence based on the dbCAN meta server search (Figure 3A and Table S3). Among the 94 GTs, 2, 1, and 4 genes predicted to encode GTs were identified uniquely based on the HMMER (dbCAN CAZyme domain HMM database), Hotpep (short conserved motifs in the PRR library database), and DIAMOND (CAZy database) searches, respectively (Table S3). Moreover, the GT2 family was prominent with 23 genes in the *F. rossica* genome (Figure 3A and Table S3). Complete genome sequences of various organisms including bacterial, archaeal, or eukaryotic organisms, reveal that a large number of GTs (about 1–2% of the total number of genes) are present in their genomes (CAZy database) [4]. Genome-wide comparisons also showed a number of genes encoding the GT2 family, suggesting that this family is a major component among GT families in most fungal species (Figure 4A and Table S3). Breton et al. [34] demonstrated that incorporation of newly discovered GT genes would increase the number of families and that not all sequences of GT were present in the public database. About 50% of the total number of GTs in the database is GT2 and GT4 families. At the time of writing (July 2019), the database comprised more than 550,978 classified and 11,654 non-classified GT sequences divided into 107 families (CAZy database). More than 170,000 sequences from various organisms were classified into the GT2 family in databases [4]. Signal peptide prediction revealed six genes comprising the signal peptides in 94 GT genes in *F. rossica* (Table S2). Signal sequence prediction of GT revealed 6 genes possessing the signal peptides in their amino acid sequences (Table S2). Most GTs are resident membrane proteins in Golgi apparatus and the endoplasmic reticulum. All GT proteins have a short N-terminal cytoplasmic tail, large C-terminal catalytic domains, and a signal-anchor domain [38]. Signal-anchor domains act as uncleavable signal peptides [39]. Thus, the predicted signal peptides in six genes likely act as signal-anchor domains. In this study, 8 genes were annotated as the GT0 family (not yet assigned to a family) in the *F. rossica* genome. GT families were defined based on significant amino acid sequence similarities [35,36]. However, previous studies have described difficulties in classifying GTs based on sequence similarity, because many GTs have divergent activities, even though their sequences are highly similar. Therefore, additional studies based on structural and mutational analyses are needed to elucidate their enzymatic characteristics.

#### 3.2.2. Carbohydrate Esterases (CEs) of the *F. rossica* Genome

Esterases act on ester bonds and are widely used as biocatalysts in biotechnology and industrial processes. [40,41]. CE represents a family of esters that generally catalyze *N*-deacylation or *O*-deacylation to remove the esters of substituted saccharides [42]. These CEs are classified into 16 families, with more than 67,000 classified (1200 non-classified) CEs in the current CAZy database (CAZy database) [4]. CEs have a variety of substrate specificities, such as specificity for acetic ester, chitin, xylan, feruloyl-polysaccharide, pectin, and peptidoglycan [43].

Our results revealed a total of 56 predicted CEs classified into 10 families in the *F. rossica* genome based on the HMMER (dbCAN CAZyme domain HMM database), Hotpep (short conserved motifs in the PRR library database), and DIAMOND (CAZy database) searches (Figure 3B and Table S3). The CE10 family was prominent with 23 CEs, and the CE4 family was the second largest family with 13 CEs in the *F. rossica* genome (Figure 3B). Genome-wide comparisons showed that the total number of CEs in *F. rossica* was similar to that found in *Flammulina* species, *Coprinopsis cinerea*, and *Schizophyllum commune*. Additionally, CE1, CE4, and CE16 families were also prominent in other basidiomycetes. However, since only 5 CE (4 CE4 and 1 CE9) and 2 CE (CE4) were found in *Cryptococcus neoformans* and *Saccharomyces cerevisiae*, respectively, the CE families were found to vary in fungal species (Figure 4B and Table S3). CAZyme prediction based on three different databases also revealed many CE10 family members in the *F. rossica* genome. However, most members of the CE10 family are reported to act on non-carbohydrate substrates [4,44]. 

Despite the large number of enzymes recently classified as CE, only a small number of members from the CE family have been biochemically and structurally analyzed, and some features of the amino acid sequences have been reported. For instance, CE families including CE1, CE4, CE5, and CE7, have been characterized as possessing the Ser-His-Asp and Gly-Xaa-Ser-Xaa-Gly conserved motifs in their amino acid sequences. In addition, CE2 and CE3 family members have the Gly-Asp-Ser-(Leu) motif rather than the Gly-Xaa-Ser-Xaa-Gly conserved motif. CE16 family members also possess the Gly-Asp-Ser-(Leu) and Ser-Gly-Asn-His motif in their amino acid sequences [45]. In the present study, we identified several CE family members containing the GXSXG conserved motifs in their amino acid sequences (Table S4). Esterase containing a Gly-Xaa-Xaa-Leu (GXXL) motif highly homologous to Class C β-lactamases was also identified [46,47]. Likewise, some CE family members were found to possess the (GXXL) motif (Table S4). Furthermore, in the present study, all genes predicted to encode CE4 family members were found to have conserved motifs such as Phe-Asp-Asp-Gly-Pro (FDDGP), in their amino acid sequences (Table S4). CE families generally catalyze *N*-deacylation or *O*-deacylation reactions of polysaccharides to promote degradation by GHs and assist biomass saccharification [48]. Therefore, an extensive range of genes encoding CE family members in the *F. rossica* genome suggests the potential for this fungus to be used in industrial applications such as biofuel production.

#### 3.2.3. Glycoside Hydrolases (GHs) of *F. rossica* Genome

GHs (glycosidases or glycosyl hydrolases, EC 3.2.1.-) are key enzymes involved in carbohydrate metabolism, which catalyze the hydrolysis of glycosidic bonds in complex carbohydrates. GHs are also common enzymes that degrade the most abundant biomass such as hemicellulose, cellulose, and starch [49,50]. GHs can be assigned to various families based on their sequence similarities.

Up to now (July 2019), the CAZy database comprised more than 664,000 classified and 10,000 non-classified GH sequences that were divided into 165 families (CAZy database) [4]. In the present study, a total of 236 GHs classified into 54 families were predicted in the *F. rossica* genome based on based on three different database searches (dbCAN, Hotpep, CAZy databases) (Figure 3C and Table S3). GH family classification also revealed that the GH16 family was prominent with 25 genes in the *F. rossica* genome (Figure 3C and Table S3). In addition, many GH16 family members were also identified in other fungal species except for some ascomycetes, including *Cordyceps militaris*, *Aspergillus nidulans*, *Trichoderma reesei*, and *S. cerevisiae* (Figure 4C and Table S3). Additionally, multiple copies of GH5 and GH18 in *F. rossica* were similar to those in other basidiomycetes. 

The GH16 family consists of agarase (EC 3.2.1.81), lichenase (EC 3.2.1.73), κ-carrageenase (EC 3.2.1.83), xyloglucan xyloglucosyltransferase (EC 2.4.1.207), endo-β-1,3-glucanase (EC 3.2.1.39), endo-β-1,3-1,4-glucanase (EC 3.2.1.6), and endo-β-galactosidase (EC 3.2.1.103), and most of these enzymes contain conserved motifs such as Glu-Xaa-Asp-Xaa-(Xaa)-Glu (EXDX[X]E). The first and last glutamic acid (E) residues of the conserved motif are characterized as a nucleophile and Brønsted acid/base, respectively, and play an important role in the catalytic activity of GH16 family enzymes [51,52,53]. All of the predicted GH16 family members in *F. rossica* showed this conserved motif, and five genes were predicted to encode GH16 contained 2 or more of the conserved motif (EXDX[X]E) (Table S4). Although not all glycosyl hydrolases have signal sequences, many GHs have signal sequences that are secreted or targeted to other cell sites including the periplasmic space or Golgi apparatus [54]. In the present study, *F. rossica* was shown to contain signal peptides in about 50% of the GH genes (98 out of 236 GH), suggesting that these GHs can be secreted (Table S2). 

GHs are essential for the processing of cellulose and xylan in plants, as well as chitins in nature (CAZy database) [4]. Cellulases (GH5, -6, -7, -8, -9, -12, -44, -45, and -48), xylanases (GH10, -11, and -30), and chitinases (GH18, -19, and -85) are known to be active against cellulose, xylose, and chitin, respectively [4,55]. *F. rossica* also contains a series of genes related to cellulase (27 genes), xylanase (6 genes), and chitinase (23 genes) in its genome sequence (Figure 3C and Table S3). Cellobiose can be converted to glucose by β-glucosidases (EC 3.2.1.21) involved in GH families such as GH1 and GH3 [4,56]. *F. rossica* also possesses GH family members including 1 GH1 and 15 GH3 in its genome (Figure 3C and Table S3).

Simultaneous actions of several GHs are necessary to effectively degrade plant cell wall complexes composed of cellulose and xylan. Recently, genome sequencing of various bacterial and fungal species has reviewed the various activities of GHs on cellulose, chitin, and xylan degradation and their potential for biotechnological applications and industrial degradation of biopolymers [55,57,58,59,60,61]. In the present study, *F. rossica* with more than 200 genes encoding various GHs showed strong potential for diverse applications, such as biotechnology and industry.

#### 3.2.4. Polysaccharide Lyases (PLs) of the *F. rossica* Genome

PLs (EC 4.2.2.-) cleave polymer chains of polysaccharides, essential cellular components of all living organisms, through a β-elimination mechanism to produce unsaturated polysaccharides [62,63]. PLs have been classified into 36 families with more than 19,600 classified and 1000 non-classified PLs in the database [4]. Our results showed that a total of 19 PLs classified into eight families were predicted in the *F. rossica* genome based on three different databases (dbCAN, Hotpep, CAZy database) searches (Figure 3D and Table S3). Among them, the PL3 family was prominent and four families, including PL4, -8, -9, and -26, consisted of only one PL (Figure 3D and Table S3). In addition, it has been found that other basidiomycetes had many PL14 family members in their genomes (Table S3). PL20 was only found in ascomycetes, whereas PL14 appeared to be specific to the Basidiomycota. Moreover, PL5, -15, and -24 family members are Basidiomycota specific; thus, the distribution of some PL family members was found to be phylum specific (Table S3) [64].

Pectate (partially branched polymers containing 1→4 linked α-d-galacturonate) or pectin (partially branched polymers containing 1→4 linked α-d-methylgalacturonate) is an acidic polysaccharide in plant cell walls [65,66,67]. The enzymes that degrade polygalacturonan (PGA) are known as pectate or pectin lyases [66]. Bacterial species mainly produce pectate lyase, whereas fungal species produce pectate lyases and pectin lyases (EC 4.2.2.10) simultaneously to act on pectin and/or pectate [67]. Pectin and/or pectate lyases have been classified into 6 PL families such as PL1, -2, -3, -9, and -10. Fungal pectate lyases (EC 4.2.2.2 and EC 4.2.2.9) were classified into PL family 1, 3, and 9 in the CAZy database [4]. The major family members of PLs were pectate lyases including PL families 1, 3, and 4 in *F. rossica* and other fungal species. However, according to this study, genes encoding PL families 2 and 10 were not found in *F. rossica* and in other fungal species genomes (Figure 3D and Table S3). Furthermore, PL family member 9 was found only in 5 *Flammulina* species, in *S. commune*, and in two ascomycetes (*A. nidulans* and *C. militaris*) but not in other fungal species (Figure 4D and Table S3).

Biochemical information on enzymes that degrade pectin or pectic bacteria in basidiomycetes is relatively scarce compared to those from other bacterial and fungal species, although genome sequencing reveals many genes encoding PL in several basidiomycetes. It is reported that *S. commune* produces high levels of pectinase and that polygalacturonase is produced at high levels, especially when cultivated in wheat bran [68]. Although no further studies were conducted in this study, there were similar numbers of genes encoding PL family members 1, 3, and 9 in the *F. rossica* genome as those in *S. commune* suggesting that *F. rossica* might be a candidate for biotechnological applications.

#### 3.2.5. Auxiliary Activities (AAs) of *F. rossica* Genome

Ligninolytic enzymes such as lytic polysaccharide monooxygenases (LPMOs) are classified into AA families, which are mainly involved in the depolymerization of non-carbohydrate structural components (lignin) of plants [6]. These AAs are classified into 16 families with more than 12,200 classified and 40 non-classified AA sequences in the current CAZy database. In addition, the AAs are presently classified into 6 families of LPMOs and 9 families of ligninolytic enzymes (CAZy database) [4]. In the present study, we identified a total of 11 AA families with 102 AAs in *F. rossica* genome sequence (Figure 3E and Table S3). Among them, AA3 (glucose-methanol-choline oxidoreductase, alcohol oxidase, aryl-alcohol oxidase/glucose oxidase, cellobiose dehydrogenase, pyranose oxidase) and AA9 (lytic polysaccharide monooxygenase) are the major members with 24 AA3 and 20 AA9 in the *F. rossica* genome, respectively (Figure 3E and Table S3). Interestingly, the total number of AAs in the *F. rossica* genome was similar to that in *C. cinerea*, but not other *Flammulina* species (Figure 4E and Table S3). In addition, our results showed that *F. rossica* had more AA1 family members in its genome compared to other basidiomycetes (Figure 4E and Table S3).

Several AA families have been shown to possess conserved motifs necessary for their activities. A laccase (EC 1.10.3.2, AA1 family) has conserved motifs (copper binding motifs) within its amino acid sequence such as His-Xaa-His, His-Xaa-His-Gly, His-Xaa-Xaa-His-Xaa-His, and His-Cys-His-Xaa^3^-His-Xaa^4^-Met/Leu/Phe [69]. Our results also showed that 7 genes predicted to encode the AA1 family contained the copper-binding motifs in their amino acid sequences (Table S4). GMC oxidoreductase protein (AA3 family) require a flavin-adenine dinucleotide (FAD) cofactor and also has a β-α-β dinucleotide binding-motif composed of Gly-Xaa-Gly-Xaa-Xaa-Gly-Xaa^18^-Glu that interacts with the FAD cofactor [70,71,72]. *F. rossica* was also found to contain 24 genes predicted to encode AA3, and 17 genes contained the β-α-β dinucleotide binding motif in their amino acid sequences (Table S4). These results indicate that the gene predicted to encode AA1 and AA3 with motifs may act as a laccase and a GMC oxidoreductase, respectively. Wood degradation by wood rotting fungi generally begins with the depolymerization of lignin, which results in further degradation of the wood polymer by highly reactive lignin radicals [73,74]. Therefore, the extensive range of enzymes belonging to AA families in the *F. rossica* genome observed in this study suggest the great potential for this fungus as a biomaterial and biofuel producer in the future.

#### 3.2.6. Carbohydrate-Binding Modules (CBMs) of *F. rossica* Genome

Amino acid sequence within a carbohydrate-active enzyme with carbohydrate-binding activity is known as CBM [75,76]. CBM generally binds to carbohydrate ligands to facilitate the catalytic activity of CAZyme. [75]. CBMs are commonly found in GHs, PLs, and GTs [77]. Moreover, CBMs are parts of scaffoldin subunit in non-hydrolytic enzymes that organizes the catalytic subunits into a cellulosome (multi-protein complexes) [76]. Enzyme complexes containing CBM in CAZymes are capable of degrading the substrate more efficiently, but it is reported that when CBM is removed from the scaffolding of cellulosomes, catalytic efficiency is reduced [76].

CBMs have been classified into 85 families with more than 180,000 classified and 550 non-classified CBMs in the CAZy database [4]. In this study, we found that a total of 21 CBMs classified into 10 families were predicted in the *F. rossica* genome based on three different database (dbCAN, Hotpep, CAZy database) searches. CBM family 13 was prominent, and six families, including CBM18, -20, -22, -35, -48, -63, and -67, were represented by only one CBM in the *F. rossica* genome (Figure 3F and Table S3). Although multiple copies of CBM13 and -50 were similar to those in other fungal species, several CBM families including CBM5, -12, -19, -32, and -43 were not found, and relatively few members of the CBM1 family were found in the *F. rossica* genome.

Additionally, the abundance of some family members was different between basidiomycetes and ascomycetes. Although CBM family 18 members of ascomycetes were found more than those in other basidiomycetes, CBM 5 and 12 families were not found in ascomycetes as well as in the *F. rossica* genome (Figure 4F and Table S3). These results are consistent with a previous study in which ascomycetes are more abundant in CBM family 18 but have less CBM5 and -12 than basidiomycetes [64]. CBMs have traditionally been regarded as essential modules of cellulases, particularly cellobiohydrolases (family GH6 and -7) [78]. Although the GH6 or GH7 members that contain CBM1 are not found in the *F. rossica* genome, some CAZyme (4 GHs and 1 CE) genes that simultaneously encode CBM suggest that CBM is required for efficient substrate degradation (Table S2).

## 4. Conclusions

This study aimed to improve the understanding of lignocellulolytic machinery in *F. rossica* and to assess its applicability in biotechnology and industry. *F. velutipes* has been previously found to convert disaccharides (cellobiose, maltose, and sucrose), trisaccharide (cellotriose), and oligosaccharide (cellotetraose) to ethanol at similar recovery rates to glucose and to convert glucose to ethanol at a similar level as *S. cerevisiae* [79,80]. This ability of *F. velutipes* is suitable for consolidated bioprocessing (CBP), which is considered an effective process for bioethanol production from lignocellulosic biomass [81,82,83]. Previously, we demonstrated that *F. velutipes*, the closest white rot fungus to *F. rossica*, is a very attractive model for bioethanol production due to the numerous genes associated with lignocellulolytic enzymes such as CAZymes [8]. In this study, we performed sequencing of the *F. rossica* genome to identify genes involved in lignocellulose degradation. As described above, various CAZyme genes were identified in the *F. rossica* genome including 102 auxiliary activities, 236 glycoside hydrolases, 94 glycosyltransferases, 19 polysaccharide lyases, 56 carbohydrate esterases, and 21 carbohydrate binding-modules. Although further studies on CAZyme genes are needed, this study suggests that *F. rossica* has great potential for future production of biomaterials such as bioenergy.

## Figures and Tables

**Table 1:** *Flammulina rossica* Genome Sequencing Statistics.

**Hiseq 2000 NGS Analysis**	Total reads (100 bp)	38,390,380
	Reads after trimming (%), >Q30	35,908,618 (93.53)
**Velvet De Novo Assembly**	Optimized Velvet hash value (kmer)	31
	Total number of contigs	15,546
	Number of contigs (>1 kb)	1843
	Contig N50 (bp)	48,718
	Length of longest contig (bp)	418,162
	Total bases in contigs (bp)	35,645,506
	Total bases in contigs (>1 kb)	33,499,878
	GC content (%)	49.79
**Gene Prediction**	Predicted gene	12,588
	Average gene length (bp)	1911
	Average protein length (aa)	510.29
	Average exon per gene	6.52
	Average exon size (bp)	234.67
	Average intron size (bp)	68.03

**Table 2:** Comparison of the Genome Characteristics of *Flammulina rossica* and other Basidiomycetes.

**Fungal Species**	*Flammulina rossica*	*F. ononidis*	*F. fennae*	*F. elastica*	*F. velutipes*	*Laccaria bicolar*	*Criptocuccus cinerea*	*Phanerochaete chrysosporium*	*Schizophyllum commune*
**Strain**	ASI4194	KACC46186	KACC46185	KACC46182	KACC42780	S238N-H82	Okayama7#130	RP78	H4-8
**Genome (Mb)**	35.6	34.5	32.4	35	35.6	64.9	37.5	35.1	38.5
**Genes**	12,588	12,269	11,591	12,536	12,218	20,614	13,544	10,048	13,181
**GC (%)**	49.79	49.76	39	49.7	48.99	46.6	51.6	53.2	56.6
**Average Gene Length (bp)**	1911	2009	1980	1973	2294	1533.0	1679.0	1667.0	1794.9
**Average Exon Size (bp)**	234.67	234.09	230.53	233.91	231.4	210.1	251.0	232.0	249.3
**Average Intron Size (bp)**	68.03	68.14	68.93	69.29	190.3	92.7	75.0	117.0	79.0

**Table 3:** Ortholog analysis of *Flammulina rossica* and other fungal species.

Fungal Species	Genes	Genes in Orthogroups (%)	Unassigned Genes (%)	Orthogroups Containing Species (%)	Number of Species-Specific Orthogroups	Genes in Species-Specific Orthogroups
*Flammulina rossica* ASI4194 *	12,588	12,024 (95.5)	564 (4.5)	7485 (58.7)	5	12
*Flammulina fennae* KACC46185 *	11,591	11,235 (96.9)	356 (3.1)	7307 (57.3)	2	4
*Flammulina ononidis* KACC46186 *	12,269	11,881 (96.8)	388 (3.2)	7572 (59.4)	1	2
*Flammulina elastica* KACC46182 *	12,536	11,987 (95.6)	549 (4.4)	7592 (59.5)	1	2
*Flammulina velutipes* KACC42780 *	12,218	10,774 (88.2)	1444 (11.8)	6679 (52.4)	4	10
*Agaricus bisporus* var. *bisporus* H97 *	10,448	9102 (87.1)	1346 (12.9)	5712 (44.8)	29	265
*Coprinopsis cinerea* okayama 7#130 *	13,356	10,837 (81.1)	2519 (18.9)	6393 (50.1)	59	329
*Cryptococcus neoformans* var. *grubii* H99 *	7826	6477 (82.8)	1349 (17.2)	4719 (37.0)	39	129
*Laccaria bicolor* S238N-H82 *	18,215	12,662 (69.5)	5553 (30.5)	6217 (48.7)	73	524
*Lentinula edodes* *	14,079	11,678 (82.9)	2401 (17.1)	6674 (52.3)	61	340
*Phanerochaete chrysosporium* RP78 *	13,602	10,443 (76.8)	3159 (23.2)	6223 (48.8)	36	265
*Schizophyllum commune* H4-8 *	13,194	10,976 (83.2)	2218 (16.8)	6214 (48.7)	59	437
*Aspergillus nidulans* FGSC-A4 **	9561	8433 (88.2)	1128 (11.8)	5709 (44.8)	4	24
*Botrytis cinerea* B05.10 **	16,389	9336 (57.0)	7053 (43.0)	6263 (49.1)	17	64
*Cordyceps militaris* CM01 **	9651	8369 (86.7)	1282 (13.3)	6131 (48.1)	5	13
*Neurospora crassa* OR74A **	10,812	8659 (80.1)	2153 (19.9)	6217 (48.7)	10	26
*Saccharomyces cerevisiae* S288C **	6002	4624 (77.0)	1378 (23.0)	3485 (27.3)	12	33
*Trichoderma reesei* QM6a **	9115	8332 (91.4)	783 (8.6)	6283 (49.3)	4	9

* Basidiomycota, ** Ascomycota.

**Table 4:** CAZymes of *Flammulina rossica* and other fungal species.

Fungal Species	CAZymes						No. of CAZyme (Annotation DB)	Total	Reference
	AA ^1^	GH ^2^	GT ^3^	CE ^4^	CBM ^5^	PL ^6^			
*Flammulina rossica* *	93	182	64	56	7	17	419 (Hmmer dbCAN)	528	This study
	61	145	53	21	5	15	300 (Hotpep)		
	39	155	72	11	11	6	294 (CAZy database)		
*Flammulina fennae* *	86	220	85	57	45	20	-	513	[10]
*Flammulina ononidis* *	87	228	87	61	40	21	-	524	[11]
*Flammulina elastica* *	82	218	89	59	42	18	-	508	[9]
*Flammulina velutipes* *	85	239	84	63	44	25	-	540	[9]
*Agaricus bisporus* *	81	174	54	33	44	9	-	395	JGI database
*Cryptococcus cinerea* *	111	195	83	60	105	16	-	570	[9]
*Laccaria bicolor* *	55	170	96	18	31	7	-	377	JGI database
*Lentinula edodes* *	82	254	85	44	61	11	-	537	[9]
*Phanerochaete chrysosporium* *	85	175	65	16	62	4	-	407	JGI database
*Schizophyllum commune* *	78	241	85	57	37	18	-	516	[9]
*Cryptococcus neoformans* *	14	97	70	5	12	4	-	202	CAZy database
*Cordyceps militaris* **	54	165	91	34	39	5	-	388	[9]
*Trichoderma reesei* **	59	210	90	32	44	5	-	440	[9]
*Saccharomyces cerevisiae* **	5	57	68	2	12	0	-	144	CAZy database
*Aspergillus nidulans* **	33	267	91	30	46	23	-	490	CAZy database
*Neurospora crassa* **	35	177	80	21	42	4	-	359	CAZy database
*Botrytis cinerea* **	77	287	119	37	89	10	-	619	CAZy database

* Basidiomycota, ** Ascomycota, ^1^ auxiliary activitie (AA), ^2^ glycoside hydrolase (GH), ^3^ glycosyltransferase (GT), ^4^ carbohydrate esterase (CE), ^5^ carbohydrate-binding module (CBM), ^6^ polysaccharide lyase (PL).

## Supplementary Materials

The following are available online at https://www.mdpi.com/2076-2607/7/10/421/s1.
